# Co-Expression of FOXP3FL and FOXP3Δ2 Isoforms Is Required for Optimal Treg-Like Cell Phenotypes and Suppressive Function

**DOI:** 10.3389/fimmu.2021.752394

**Published:** 2021-10-19

**Authors:** Yohei Sato, Jessica Liu, Esmond Lee, Rhonda Perriman, Maria Grazia Roncarolo, Rosa Bacchetta

**Affiliations:** ^1^ Department of Pediatrics, Division of Hematology, Oncology, Stem Cell Transplantation and Regenerative Medicine, Stanford University School of Medicine, Stanford, CA, United States; ^2^ Institute for Stem Cell Biology and Regenerative Medicine, Stanford University School of Medicine, Stanford, CA, United States; ^3^ Center for Definitive and Curative Medicine (CDCM), Stanford University School of Medicine, Stanford, CA, United States

**Keywords:** Foxp3, alternative splicing, gene editing (CRISPR/Cas9), gene therapy, suppressive function, regulatory T cell (Treg)

## Abstract

FOXP3 is the master transcription factor in both murine and human FOXP3^+^ regulatory T cells (Tregs), a T-cell subset with a central role in controlling immune responses. Loss of the functional Foxp3 protein in *scurfy* mice leads to acute early-onset lethal lymphoproliferation. Similarly, pathogenic FOXP3 mutations in humans lead to immunodysregulation, polyendocrinopathy, enteropathy, and X-linked (IPEX) syndrome, which are characterized by systemic autoimmunity that typically begins in the first year of life. However, although pathogenic *FOXP3* mutations lead to overlapping phenotypic consequences in both systems, FOXP3 in human Tregs, but not mouse, is expressed as two predominant isoforms, the full length (FOXP3FL) and the alternatively spliced isoform, delta 2 (FOXP3Δ2). Here, using CRISPR/Cas9 to generate FOXP3 knockout CD4^+^ T cells (FOXP3KO^GFP^ CD4+ T cells), we restore the expression of each isoform by lentiviral gene transfer to delineate their functional roles in human Tregs. When compared to FOXP3FL or FOXP3Δ2 alone, or double transduction of the same isoform, co-expression of FOXP3FL and FOXP3Δ2 induced the highest overall FOXP3 protein expression in FOXP3KO^GFP^ CD4+ T cells. This condition, in turn, led to optimal acquisition of Treg-like cell phenotypes including downregulation of cytokines, such as IL-17, and increased suppressive function. Our data confirm that co-expression of FOXP3FL and FOXP3Δ2 leads to optimal Treg-like cell function and supports the need to maintain the expression of both when engineering therapeutics designed to restore FOXP3 function in otherwise deficient cells.

## Introduction

Regulatory T cells (Tregs) are defined as a CD4^+^ CD25^+^ CD127^-^ population and characterized by their suppressive function in both human and mice (1, 2). *Forkhead Box P3* (*FOXP3*) is an essential transcription factor for the development of functional Tregs in both systems (3, 4). The role of FOXP3 in regulating the suppressive function of human Tregs is exemplified by loss-of-function *FOXP3* mutations, which result in primary Treg dysfunction and effector T cell (Teff) abnormalities, leading to the monogenic autoimmune disease, IPEX syndrome (5–10). Similarly, mutation of *Foxp3* (Foxp3^SF^) in mice results in fatal systemic lymphoproliferation and rapid wasting disease. The “scurfy” mice, which has many similarities to IPEX syndrome, can be rescued by wild-type (WT) Treg transfer (11).

In human T cells, FOXP3 is expressed as two predominant isoforms, the full-length isoform, FOXP3FL, and an alternatively spliced isoform, FOXP3Δ2 that lacks exon 2. Two other isoforms, FOXP3Δ2Δ7 and FOXP3Δ7, have also been described in human lymphocytes and epithelial cells, but unlike FOXP3FL and FOXP3Δ2, neither appears to play a role in Treg suppressive activity (12). Therefore, the need to maintain their expression when restoring a dysregulated immune system, such as that in IPEX patients, is not clear (13, 14). In contrast to human T cells, mice express only FOXP3FL (15). The fact that FOXP3Δ2 has not been detected in mouse hints at key evolutionary differences in T cell lineage development between the two species (16). Importantly, clearly defining the differential or complementary roles of FOXP3FL and FOXP3Δ2 in human health and disease has been challenging, due to limitations in experimental tools, including humanized mouse models, to cleanly characterize the function of each isoform, a lack of antibodies that can specifically detect the FOXP3Δ2 isoform (anti-FOXP3 antibody clone 150D/E4 binds to exon 2 and can detect its absence), and no currently identified strategies that can accurately replicate the endogenous alternative splicing of FOXP3 exon 2 (17). Notably, a recent paper reported a family with a *FOXP3* mutation (c.305delT: p.Phe102fs) that led to loss of FOXP3FL but maintained the expression of FOXP3Δ2. Interestingly, affected males reached adulthood suggesting a level of immune functionality, but they did develop autoimmune manifestations including type 1 diabetes, inflammatory bowel disease, and psoriatic arthritis (17). These findings indicate that Tregs may be able to differentiate with FOXP3Δ2 in the absence of FOXP3FL, but that expression of only FOXP3Δ2 may lead to impaired suppressive function which cannot be rescued from the absence of FOXP3FL. Collectively, the data hint that both FOXP3FL and FOXP3Δ2 isoforms may be necessary for Tregs to fully acquire suppressive functionality.

In recent years, we have established several *FOXP3* gene transfer approaches mainly for clinical translational purposes but which have also provided fundamental findings, clarifying the role of FOXP3 in human T cells (18). We have demonstrated that lentiviral-mediated gene transfer of FOXP3FL under a constitutive promoter in Teff cells is sufficient to convey suppressive function, upregulate several key Treg molecules, and suppress others so that the transduced Teff cells become very similar to Tregs (“CD4^LVFOXP3^ Treg-like cells”). Additionally, CRISPR/Cas9 *FOXP3* gene editing in which we inserted either FOXP3FL or FOXP3Δ2 at the FOXP3 locus in Teff or Tregs revealed comparable activities for the two isoforms, with each inducing WT restoration of function in Teff cells, but only suboptimal repair of function in Tregs, suggesting that the simultaneous expression of both isoforms is a requirement for optimal Treg development and function (19). Further, although other published gene transfer studies have explored the roles of FOXP3FL and FOXP3Δ2, these experiments were performed in T cells expressing endogenous *FOXP3*, therefore never clearly dissecting the impact of each isoform, or both (15).

Here, using our established Teff to Treg “conversion system,” we elucidate the roles for each isoform in conveying Treg-cell features and functions. Using CRISPR/Cas9, we have first made CD4^+^ T cells knocked out (KO) for endogenous *FOXP3* (FOXP3KO^GFP^ CD4+ T cells). The FOXP3KO^GFP^ CD4+ T cells were then transduced with lentiviral vectors expressing FOXP3FL and/or FOXP3Δ2. Each isoform was cloned into lentiviral vectors (LV-) co-expressing the truncated *Epithelial Growth Factor Receptor* (EGFR) or *Nerve Growth Factor Receptor* (NGFR) surface marker, thus enabling both simultaneous transduction and tracking of the expression of each isoform, as well as delineation of their functional roles. Our data demonstrates that co-transduction of both FOXP3FL and FOXP3Δ2 isoforms, in comparison to each alone, is optimal for the conversion of FOXP3KO^GFP^ CD4+ T cells into functional Treg-like cells *in vitro.* These findings suggest that both isoforms play key roles in Treg-cell function and provide important considerations for the design of gene engineering strategies in stem cell progenitors aimed at restoring FOXP3 expression and function across different T cell lineages.

## Materials and Methods

### Cell Isolation and *In Vitro* Culture

HEK293T/17 cells were purchased from ATCC. HEK293T/17 cells were cultured with Dulbecco’s modified Eagle’s medium (DMEM, Life Technologies) supplemented with 10% fetal bovine serum (FBS, Gibco). MT-2 cells are human adult Treg-cell lines immortalized with HTLV-1 and were kindly provided by Dr. Donald B. Kohn (UCLA) (20, 21). MT-2 cells maintain a very high expression of FOXP3 but have the ability to proliferate without TCR-mediated restimulation, and have been elsewhere characterized (22). MT-2 cells were cultured with Roswell Park Memorial Institute medium (RPMI, Life Technologies) supplemented with 10% FBS. Mycoplasma testing (MycoAlert Quick Mycoplasma Detection Kit, Lonza) was done routinely with mycoplasma contamination not detected during the study period.

Buffy coats from healthy male donors were obtained from the Stanford Blood Center. Peripheral blood mononuclear cells (PBMCs) were separated from the Buffy coat by density gradient centrifugation using Ficoll Paque (GE Healthcare Science). CD4^+^ T cells were enriched from PBMCs by negative selection (Stem Cell Technologies). CD4^+^ T cells were cultured by X-VIVO 15 media supplemented with gentamicin (Lonza) and 5% human serum from pooled male AB plasma (Sigma-Aldrich) with the presence of 100 U/ml of recombinant human (rh) IL-2 (PeproTech). Human expanded Tregs were generated according to previous publications (23, 24). Briefly, CD4^+^ T cells were activated by CD3/CD28 polyclonal stimulation and cultured in the presence of rapamycin (100 nM). Expanded Tregs were harvested and analyzed on day 21 (suppression assay) and day 28 (phenotype).

### *FOXP3* Gene Disruption by CRISPR/Cas9-Mediated Homologous Recombination

The *FOXP3* gene was disrupted in CD4^+^ T cells by CRISPR/Cas9-mediated homologous recombination. Freshly isolated CD4^+^ T cells were activated overnight by plate-bound 10 μg/ml of anti-CD3 antibody (eBioscience) and 1 μg/ml of anti-CD28 antibody (eBioscience). A ribonucleoprotein (RNP) complex was made by incubating 15 μg of Cas9 protein (Alt-R^®^ S.p. Cas9 Nuclease V3, Integrated DNA Technologies) with 8 μg of a small guide RNA (sgRNA; TriLink BioTechnologies) targeting FOXP3 with the following sequence: 5′-2′OMe[A(ps)G(ps)G(ps)]ACCCGAUGCCCAACCCCGUUUUAGAGCUAGAAAUAGCAAGUUAAAAUAAGGCUAGUCCGUUAUCAACUUGAAAAAGUGGCACCGAGUCGGUGCUUU 2′OMe (U(ps) U(ps)U)-3′ where ps = phosphorothioate, 2′OMe = 2′-O-methyl, at room temperature for 15 min. The RNP complex, together with an AAV6 vector (multiplicity of infection (MOI) of 100,000) containing FOXP3 homologous arms and GFP expression cassette under the PGK promoter (Vigene), was transduced into CD4^+^ T cells using the Amaxa 4D-Nucleofector (Lonza). The GFP-positive population was sorted by FACS 4–5 days after transduction.

### LV Vector Construct and Transduction

Vectors LV-FOXP3FL (FOXP3FL-NGFR) and LV-FOXP3Δ2 (FOXP3Δ2-NGFR) were kindly provided by Dr. Megan K Levings (University of British Columbia). Vectors FOXP3FL-EGFR and FOXP3Δ2-EGFR were generated from FOXP3FL-NGFR and FOXP3Δ2-NGFR by restriction enzyme digestion (AgeI and BlpI, New England Biolabs Inc.) to remove NGFR, then EGFR was inserted using a DNA ligase (Mighty Mix DNA Ligation Kit, ClonTech; **Figure S1A**). Control vectors expressing only NGFR or EGFR were also constructed.

Lentivirus was produced as previously described (18, 25, 26). Briefly, each vector together with helper plasmids (gag/pol, REV, VSVG, and pAdVAntage) were transiently co-transfected into HEK293T/17 cells by Mirus-LT1 reagent (Mirus Bio LLC, Madison, WI). The supernatant was collected 30 h after transfection and concentrated by ultracentrifugation. The titer of lentivirus was determined by serial dilution of 293T cells.

For the single transduction of each LV construct, FOXP3KO^GFP^ CD4+ T cells were activated overnight by TransAct (Miltenyi Biotec) with 100 U/ml rhIL-2 (PeproTech) and 10 ng/ml rhIL-7 (PeproTech). On day 1, lentivirus was added to the activated T cells at the MOI of 50. Cells were split every 2–3 days. On days 9–10, the transduced FOXP3KO^GFP^ CD4+ T cells were enriched using the MACSelect LNGFR System (NGFR; for vector constructs co-expressing NGFR). For the sequential transduction, FOXP3KO^GFP^ CD4+ were sequentially transduced with a second LV construct after the activation and enriched by the MACS PE beads with an anti-human EGFR antibody conjugated with PE (EGFR; for vector constructs expressing EGFR) (Miltenyi Biotec).

We have confirmed isoform expression in HEK293T/17 cells by transducing lentiviral vectors expressing 1) FOXP3FL (NGFR); 2) FOXP3Δ2 (EGFR), or 3) FOXP3FL (NGFR)+FOXP3Δ2 (EGFR) (**Figures S1B, D**). FACS analysis confirmed that all three conditions gave a robust FOXP3 expression. Notably, the HEK293T/17 cells double transduced with both FOXP3 isoforms gave the highest FOXP3 expression (**Figures S1C, E**).

### Vector Copy Number

Genomic DNA was extracted from 1 × 10^6^ transduced FOXP3KO^GFP^ CD4+ T cells by DNeasy Blood and Tissue Kit (Qiagen). Lentiviral copy numbers were measured by *Woodchuck Hepatitis Virus Posttranscriptional Responsive Element (WPRE)* qPCR, as previously described (27). Primer sequences for copy number qPCR were *WPRE* (forward primer, 5′-CCGTTGTCAGGCAACGTG-3′; reverse primer, 5′-AGCTGACAGGTGGTGGCAAT-3′; probe, 5′-FAM TGCTGACGCAACCCCCACTGGT-TAMRA-3) and *ALB* (forward primer, 5′-TGAAACATACGTTCCCAAAGAGTTT-3′; reverse primer, 5′-CTCTCCTTCTCAGAAAGTGTGCATAT-3′; probe, -FAM- TGCTGAAACATTCACCTTCCATGCAGATAMRA-3′). The copy number using *WPRE/ALB* primers was determined using 10 ng of genomic DNA. VCN was calculated by *WPRE*/*ALB* copy number and shown as copy number per genome.

### RT-PCR

RNA was extracted from 1 × 10^6^ FOXP3KO^GFP^ MT-2 or CD4+ T cells by RNeasy Mini Kit (Qiagen). One hundred nanograms of total RNA was used to synthesize cDNA using SuperScript IV VILO (Thermo Fisher Scientific). RT-PCR was done using 1 μl of synthesized cDNA amplified by PrimeSTAR GXL DNA Polymerase (Takara Bio) as described in (15). Primer sequences were *FOXP3* (forward primer, 5′-TTCCTTGGCCCTTGGCCCATC-3′; reverse primer, 5′-CATTTGCCAGCAG TGGGTAGGA-3′) and *ACTB* (forward primer, 5′-CCTCGCCTTTGCCGATCC-3′; reverse primer, 5′-GGATCTTCATGAGGTAGTCAGTC-3′). PCR products were analyzed by 2% agarose gel electrophoresis.

### qPCR

Total RNA was extracted from 1 million CD4^+^ T cells by RNeasy Plus Mini Kit (Qiagen). One hundred nanograms of RNA was used to synthesize cDNA by SuperScript IV VILO (Thermo Fisher Scientific, Waltham, MA). Q-PCR was done by TaqMan Gene Expression Assay and TaqMan Universal Master Mix II (Thermo Fisher). Gene expression was calculated by ΔCT where ΔCT = CT_target_ - CT_housekeeping_. The internal control housekeeping gene is *HPRT* (Hs99999909_m1). The expressions of the target gene were measured by q-PCR primers (TaqMan Gene Expression Assay) targeting *FOXP3* (Hs01085834_m1), *NGFR* (Hs00609976_m1), *EGFR* (Hs01076088_m1), *GARP* (Hs00194136_m1), and *LAP* (Hs00998133_m1).

### Flow Cytometry

CD4^+^ T cells were resuspended in flow cytometry (FACS) buffer (PBS supplemented with 0.5% BSA and 2 mM EDTA) and stained with antibody cocktails (CD4, CD25, CD127, NGFR, and EGFR) for 30 min. After surface staining, intracellular staining for FOXP3 and GFP was done using the Foxp3/Transcription Factor Staining buffer set (eBioscience). Antibodies used are listed in **Table S1**. Data were acquired by (BD Biosciences) and analyzed by FlowJo 10.4 software (FlowJo LLC).

### Immunoblotting

Immunoblotting was done to analyze FOXP3 isoform expression. Briefly, total protein was extracted from transduced FOXP3KO^GFP^ MT-2 or CD4+ T cells by RIPA buffer (Life Technologies) and protein concentration was measured by Pierce BC Protein Assay Kit (Thermo Fisher Scientific). Ten micrograms of protein per condition was loaded on the 4%–15% gradient SDS-PAGE gel (Bio-Rad) and transferred to a PVDF membrane (Bio-Rad). The primary antibody (Anti-FOXP3 antibody Rabbit, Cell Signaling Technologies) was added overnight at the concentration of 1:1,000. The secondary antibody (Anti-Rabbit IgG antibody-HRP conjugated, Cell Signaling Technologies) was added for 1 h at the concentration of 1:10,000. Antibodies used are listed in **Table S2**. Chemiluminescent substrate (Clarity Western ECL Substrate, Bio-Rad) was added to the membrane, and the signal was captured using the ChemiDoc XRS+ system.

### ELISA

Transduced FOXP3KO^GFP^ CD4+ T cells were cultured at the density of 1 × 10^6^ cells/ml in a round-bottom 96-well plate and activated by Dynabeads Human T-cell Activator CD3/CD28 (Gibco) at a 1:25 bead: cell ratio. Culture supernatant was collected at 24 h (IL-2) and 72 h (IL-4, IFN-γ, IL-17A, IL-22) after stimulation. Cytokine concentrations, including IL-2, IL-4, and IFN-γ; were measured by Human OptEIA ELISA Kit (BD Biosciences). Cytokine concentrations, including IL-17A and IL-22 concentrations, were measured by Human DuoSet ELISA Kit (R&D).

### Suppression Assay

Suppression assay for transduced FOXP3KO^GFP^ CD4+ T cells was done according to the previous publication (18). Briefly, responder CD4^+^ T cells (50,000 cells) labeled by CellTrace CFSE (Life Technologies) and transduced FOXP3KO^GFP^ suppressor cells (12,500–50,000 cells) labeled by CellTrace Violet (Life Technologies) were cocultured at different concentrations (1:0.25–1:1) after activation by Dynabeads Human T-cell Activator CD3/CD28 (Gibco) at the 1:25 bead: cell ratio. Ninety-six hours after stimulation, proliferations of responder CD4^+^ T cells were counted by FACS.

### Statistical Analysis

GraphPad Prism Software ver8.0 (GraphPad software) was used for statistical analyses. All statistical analyses were performed with the two-tailed Student t-test or Mann–Whitney U-test. One-way ANOVA followed by Tukey’s multiple-comparison tests was performed when comparing multiple conditions. p values <0.05 were considered as significant. Data are shown as mean ± SEM.

## Results

### FOXP3 Expression Can Be Restored in FOXP3KO^GFP^ MT-2 Cells by LV-Mediated FOXP3 Isoform Gene Transfer

**Figure 1:**
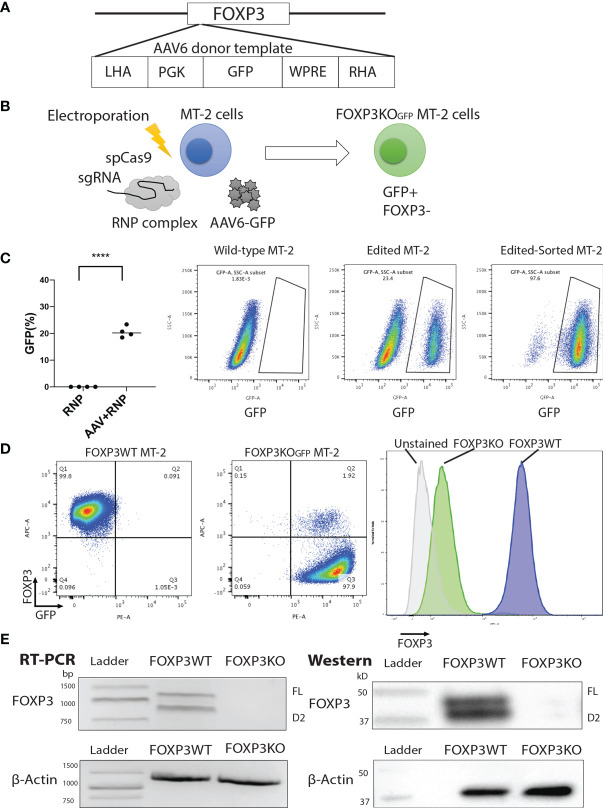
FOXP3KO^GFP^ MT-2 cells can be generated by CRISPR/Cas9-mediated homologous recombination. The *FOXP3* locus of MT-2 cells was knocked out by CRISPR/Cas9-mediated homologous recombination. **(A)** Design of the PGK-*GFP* AAV6 donor template, left homology arm (LHA), Woodchuck Hepatitis Virus Posttranscriptional Regulatory Element (WPRE), and right homology arm (RHA). **(B)** *FOXP3* Editing strategy of MT-2 cells. **(C)** *FOXP3* editing efficiency of MT-2 cells (n = 4). **(D)** Representative FACS staining of FOXP3KO^GFP^ MT-2 cells. **(E)** RT-PCR and immunoblotting of FOXP3KO^GFP^ MT-2 cells. ****P < 0.0001.

To first test and optimize the FOXP3KO and ectopic *FOXP3* gene re-expression strategy, we used the MT-2 cell-line, which grows easily in culture and aberrantly expresses FOXP3, in an activation-independent manner. We disrupted the FOXP3 open reading frame in MT2 cells using CRISPR/Cas9 and an sgRNA targeting the *FOXP3* locus. A GFP open reading frame (expressed *via* the constitutive PGK promoter and stabilized by the WPRE element which follows) was inserted at the cut site, after delivery to cells using an AAV6 vector; GFP expression was used to purify the edited cells (**Figures 1A, B** **)**. Using this strategy, we generated GFP-positive FOXP3KO MT-2 cells (FOXP3KO^GFP^ MT-2: targeting efficiency 20.6 ± 1.04%, MEAN ± SEM, n = 4) which were enriched by FACS sorting (**Figures 1C, D** **)** to produce a pure population of the edited MT-2 cells. RT-PCR and immunoblotting (**Figure 1E**) confirmed that the FOXP3WT MT-2 cells highly expressed both isoforms of FOXP3, prior to gene editing, but that neither were detectable in the sorted GFP+ MT-2 cells, following the CRISPR/Cas9-mediated disruption of the endogenous *FOXP3* gene.

**Figure 2:**
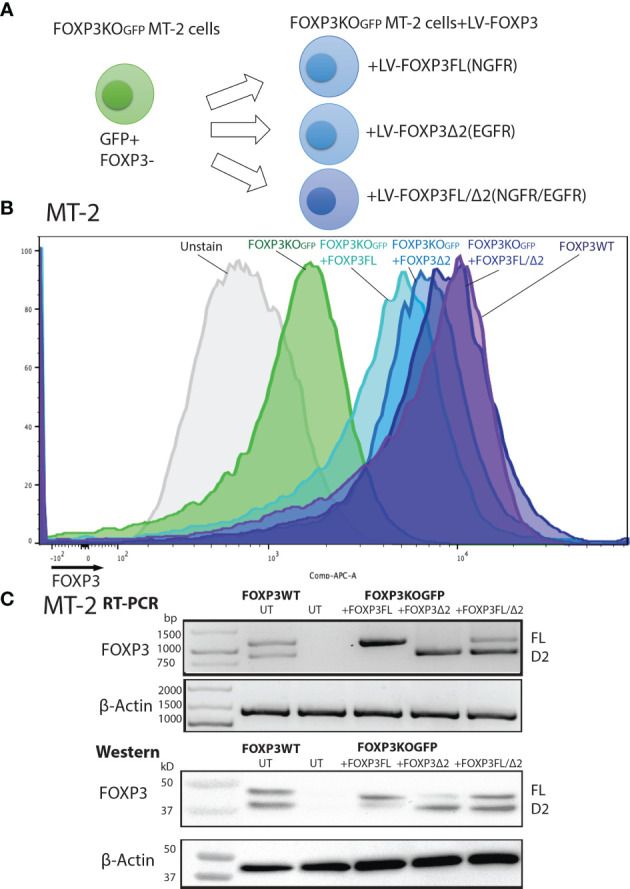
FOXP3KO^GFP^ MT-2 cells transduced with FOXP3 isoforms. FOXP3KO^GFP^. MT-2 cells were transduced with FOXP3FL and/or FOXP3Δ2. **(A)** LV transduction strategy of FOXP3KO^GFP^ MT-2 cells. **(B)** FACS staining of FOXP3 knockout MT-2 cells. **(C)** RT-PCR and immunoblotting of FOXP3KO^GFP^ MT-2 cells.

FOXP3KO^GFP^ MT-2 cells were then transduced with lentiviral vectors expressing FOXP3FL-NGFR or FOXP3Δ2-EGFR, or both, and protein expression examined by FACS analysis (**Figure 2A** and **Figures S2A, B**). Single transduction of either FOXP3FL or FOXP3Δ2 restored FOXP3 expression in FOXP3KO^GFP^ MT-2 cells. Notably, FOXP3 expression from cells expressing FOXP3FL or FOXP3Δ2 was not as high as that of FOXP3WT MT-2 cells. In contrast, we obtained an almost full restoration of FOXP3 expression, when both isoforms (FOXP3FL+ FOXP3Δ2) were transduced (**Figures 2B, C** **)**. We also transduced the two isoforms into FOXP3WT MT-2 cells (i.e., expressing endogenous FOXP3) but saw no increase in FOXP3 expression, suggesting that expression levels of this gene are tightly regulated or that FOXP3 expression cannot be increased beyond the endogenous level (**Figures S3A–C**). Taken together, these data show that LV-mediated *FOXP3* gene transfer can restore FOXP3 expression in FOXP3KO^GFP^ MT-2 cells and that dual transduction of both isoforms is feasible and traceable with the different marker genes. Those results support our strategy for further testing in CD4+ T cells.

### Single FOXP3 Isoform Ectopic Expression in FOXP3KO^GFP^ CD4^+^ T Cells

**Figure 3:**
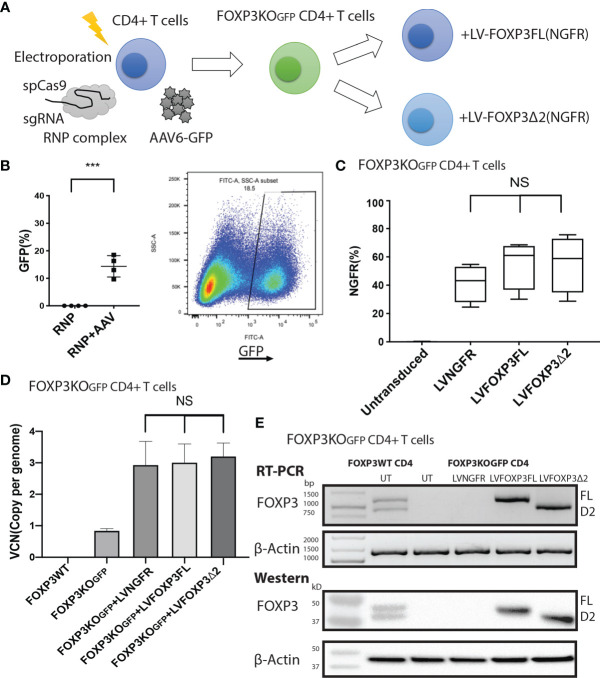
FOXP3KO^GFP^ CD4+ T cells can be transduced with FOXP3 isoforms. FOXP3KO^GFP^ CD4+ T cells were transduced with FOXP3FL (NGFR) or FOXP3δ2 (NGFR). **(A)** LV transduction strategy of FOXP3 knock-out CD4+ T cells. **(B)** Editing efficiency of CD4+ T cells (n = 4). **(C)** Transduction efficiency of FOXP3KO^GFP^ CD4+ T cells (n = 4). **(D)** VCN analysis of FOXP3KO^GFP^ CD4+ T cells transduced with FOXP3 isoforms (n = 4). **(E)** RT-PCR and immunoblotting of FOXP3KO^GFP^ CD4+ T cells transduced with FOXP3 isoforms. ***P < 0.001, NS, not significant.

To assess the roles of each FOXP3 isoform alone, or together, in the conversion of Teff into Tregs, we used our previously established robust experimental system, in which transduction of LV-FOXP3FL converts CD4+ T cells into Treg-like cells (25, 26). To use this experimental system, we first disrupted the endogenous *FOXP3* gene and inserted GFP at the cut site of CD4+ T cells (FOXP3KO^GFP^ CD4+ T cells), as was done in the MT-2 cells. We generated GFP-positive FOXP3 KO CD4+ T cells (targeting efficiency 14.4 ± 1.93%, MEAN ± SEM, n = 4) and enriched the GFP+ cells by FACS sorting (**Figures 3A, B** **)**. The FOXP3KO^GFP^ CD4+ T cells were then used to test the functional impact of LV-FOXP3FL or LV-FOXP3Δ2 gene transfer. The efficiency of LV transduction into the FOXP3KO^GFP^ CD4+ T cells was considerably lower than our previous experience using similar vectors in primary CD4+ T cells that have not undergone CRISPR/Cas9 editing (18). However, selection of the transduced cells by detection of the co-expressed NGFR or EGFR surface markers allowed us to purify the transduced cells and evaluate the expression and impact of each isoform on cell functions (**Figure 3C**). Notably, there was no significant difference in the LV vector copy number (VCN) between the FOXP3FL- and FOXP3Δ2-transduced cells (FOXP3FL 3.01 ± 0.59 copy/cell, FOXP3Δ2 3.21 ± 0.42 copy/cell, NGFR 2.93 ± 0.37 copy/cell, MEAN ± SEM, n = 4) (**Figure 3D**). At the same time, it was shown that FOXP3KO^GFP^ CD4+ T cells have a VCN of 0.85 ± 0.03 copy/cell, which corresponds to the WPRE element inserted in the FOXP3 locus. Further, RT-PCR and immunoblotting demonstrated equivalent FOXP3 mRNA and protein in cells transduced with each isoform (**Figure 3E**). Moreover, as expected, activation-induced FOXP3 expression observed in the FOXP3WT CD4+ T cells was not detected in the FOXP3KO^GFP^ CD4+ T cells. These data do confirm that LV-mediated gene transfer of either FOXP3FL or FOXP3Δ2 into FOXP3KO^GFP^ CD4+ T cells restores the expression of each FOXP3 isoform.

**Figure 4:**
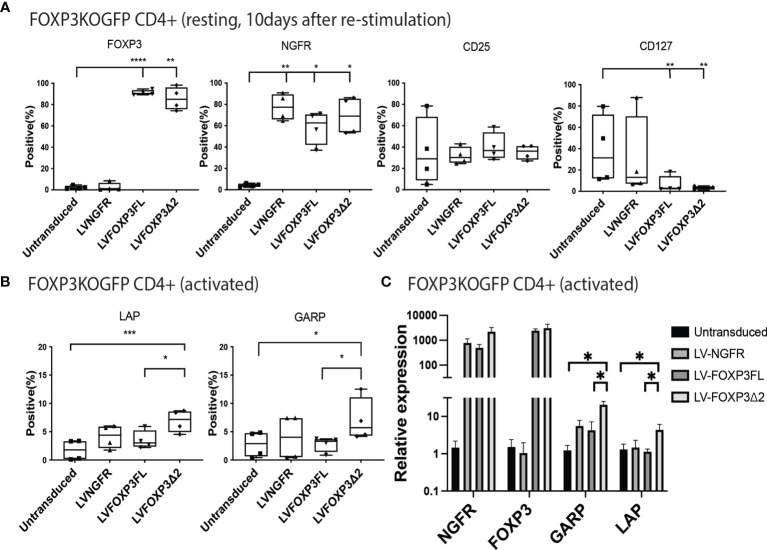
Single transduction of different isoforms provides a Treg-like phenotype in FOXP3KO^GFP^ CD4+ T cells. **(A, B)** FACS staining of FOXP3KO^GFP^ CD4+ T cells transduced with single FOXP3 isoforms (n = 4). **(C)** qPCR analysis of FOXP3KO^GFP^ CD4+ T cells transduced single FOXP3 isoforms (n = 4). *P < 0.05, **P < 0.01, ***P < 0.001, ****P < 0.0001.

**Figure 5:**
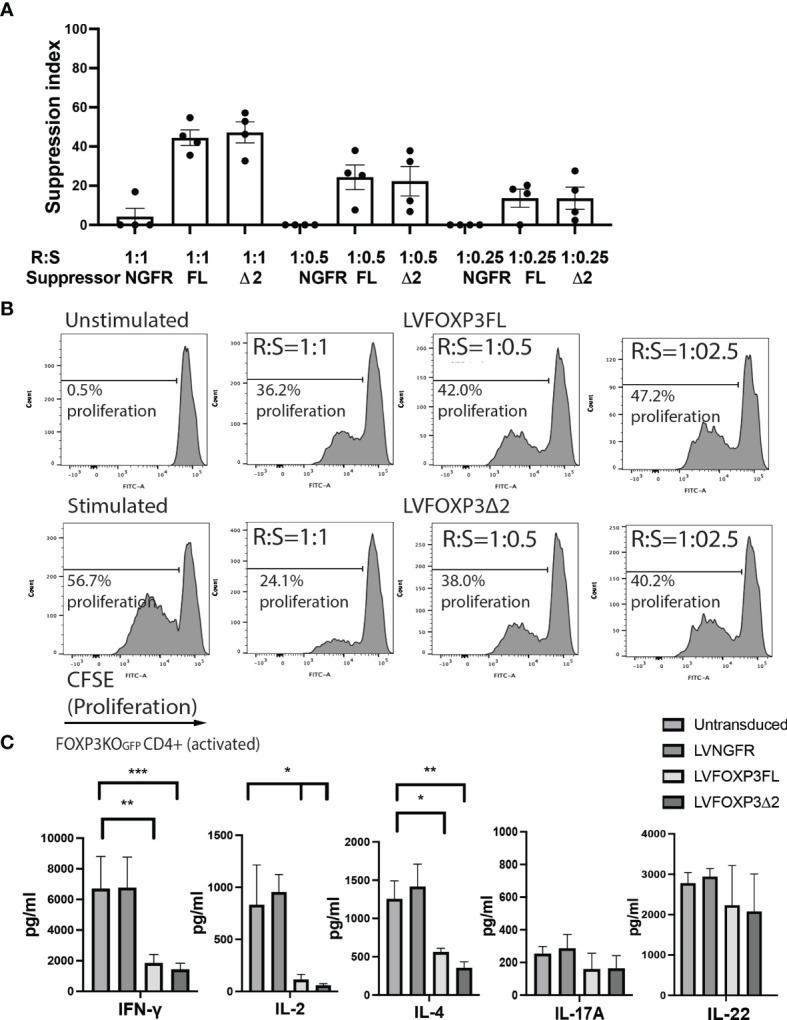
Single transduction of different isoforms provides Treg-like function in FOXP3KO^GFP^ CD4+ T cells. **(A, B)** Suppressive function of FOXP3KO^GFP^ CD4+ T cells transduced with single FOXP3 isoforms (n = 4). **(C)** Cytokine production profile of FOXP3KO^GFP^ CD4+ T cells transduced with FOXP3 isoforms (n = 4). *P < 0.05, **P < 0.01, ***P < 0.001.

In addition to restored FOXP3 expression, FOXP3KO^GFP^ CD4+ T cells transduced with LV-FOXP3FL or LV-FOXP3Δ2 displayed a Treg-like phenotype (CD25high CD127low FOXP3positive), which was comparable to that of expanded Tregs, evaluated as a control (**Figures 4A** and **S4A**), and data are summarized in **Table 1**. Further and consistent with the suggested role for FOXP3Δ2 in inducing the expression of LAP (latency-associated peptide) and GARP (glycoprotein A repetitions predominant) (28–31), we found that these Treg-related proteins were significantly higher in activated FOXP3δ2-transduced FOXP3KO^GFP^ CD4+ T cells than in FOXP3FL-transduced FOXP3KO^GFP^ CD4+ T cells (**Figures 4B** and **S5A, B**). LAP and GARP mRNA expressions were significantly higher in the FOXP3Δ2-transduced FOXP3KO^GFP^ CD4+ T cells than in FOXP3FL-transduced FOXP3KO^GFP^ CD4+ T cells (**Figure 4C**). FOXP3KO^GFP^ CD4+ T cells transduced with either FOXP3 isoform had a comparable suppressive activity in inhibitions of primary CD4^+^ T cell proliferation upon activation (suppression index FOXP3FL 44.5 ± 4.0 (R:S = 1:1), FOXP3Δ2 47.2 ± 5.3 (R:S = 1:1), MEAN ± SEM, n = 4) (**Figures 5A, B** **)**. The suppression level of the FOXP3KO^GFP^ CD4+ T cells transduced with either FOXP3 isoform was lower than that observed with expanded WT Tregs (suppression index expanded WT Tregs 89.9 ± 1.0 (R:S = 1:1), MEAN ± SEM, n = 4) (**Figure S4B**), expanded WT Tregs in the literature (32), and also lower than the previously reported WT CD4+ T cells converted into Treg with FOXP3 FL only (25, 26). Finally, and consistent with the known FOXP3-mediated suppression of specific cytokines, the activated FOXP3KO^GFP^ CD4+ T cells transduced with each isoform had a significantly reduced production of IL-2, IL-4, and IFN-γ when compared to untransduced or LV-NGFR-transduced cells (**Figure 5C**). Further, although not statistically significant, IL-17A and IL-22 expression also trended down, when compared to untransduced or LV-NGFR-transduced cells. These results indicate that the LV-mediated transfer of either FOXP3FL or FOXP3Δ2 into FOXP3KO^GFP^ CD4+ T cells can induce a Treg-like phenotype including suppression of cytokine production but that the suppressive function induced by each isoform alone is significantly less than that observed in expanded WT Tregs.

**Table 1:** Summary of Treg-like characteristics of FOXP3KO^GFP^ CD4+ T cells transduced with FOXP3FL/Δ2.

	FOXP3WT	FOXP3KOGFP	FOXP3KOGFP	FOXP3KOGFP	FOXP3KOGFP	FOXP3KOGFP	FOXP3KOGFP	FOXP3KOGFP	FOXP3KOGFP	Expanded
			+LVNGFR	+LVFOXP3FL	+LVFOXP3d2	+LVNGFR/EGFR	+LVFOXP3FL/FL	+LVFOXP3d2/d2	+LVFOXP3FL/d2	Tregs
Number of transduction	0	0	1	1	1	2	2	2	2	0
VCN (copy/genome)	0.00	0.85	2.94	3.01	3.21	3.92	4.08	4.09	4.22	NA
Phenotype (FACS)										
CD25 (%)	22.8	35.4	32.0	28.3	27.0	57.6	82.8	87.1	85.6	84.2
CD127 (%)	62.9	38.5	30.2	6.3	3.1	51.7	14.3	7.3	6.6	19.3
FOXP3 (%)	2.4	1.1	2.7	91.2	85.5	0.0	90.2	88.1	94.4	97.3
FOXP3 (MFI)	115.1	8.6	13.1	824.5	924.0	42.8	1888.0	1899.0	2580.0	NA
GARP (%)	2.0	2.8	2.0	2.8	7.0	1.8	4.4	14.3	14.7	NA
LAP (%)	2.0	1.8	3.4	3.5	6.4	2.2	2.4	5.4	9.0	NA
Suppression index										
1:1	NA	NA	4.2	44.5	47.2	11.0	57.5	60.1	88.0	89.9
1:0.5	NA	NA	0.0	24.4	22.3	3.1	39.7	46.8	51.8	86.1
1:0.25	NA	NA	0.0	13.6	18.6	1.8	23.7	16.7	48.3	57.5
Cytokine (ELISA, pg/ml)										
IL-2	470.8	830.3	951.6	112.4	56.9	732.5	121.4	119.4	98.8	NA
IL-4	1485.0	1254.0	1413.0	558.9	352.6	1453.0	521.0	532.6	448.7	NA
IFN-γ	7171.0	6695.0	6756.0	1836.0	1421.0	6136.0	916.5	749.0	518.6	NA
IL-17A	353.7	253.5	285.8	157.8	162.8	635.4	250.4	233.2	143.9	NA
IL-22	2897.0	2869.0	3036.0	2303.0	2142.0	2960.0	1475.0	1658.0	383.1	NA

NA, not applicable.

### Co-Expression of FOXP3FL and FOXP3Δ2 Provides Optimal Treg-Like Phenotype and Function in FOXP3KO^GFP^ CD4+ T Cells

**Figure 6:**
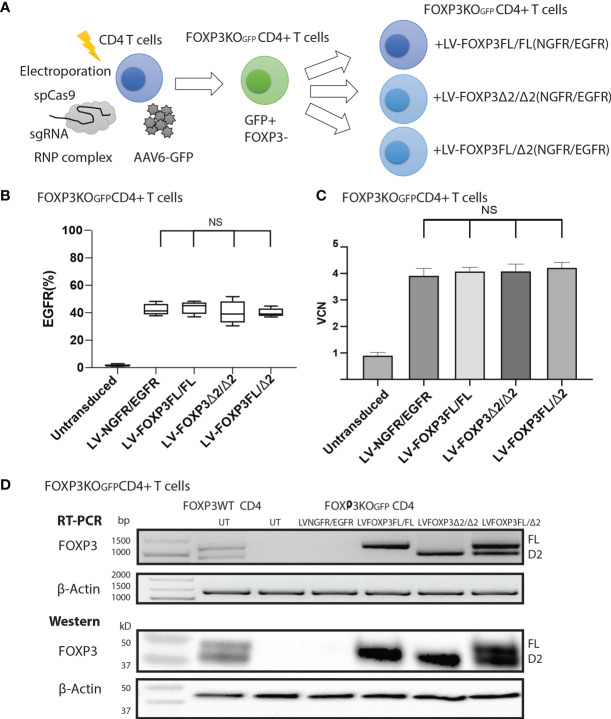
FOXP3KO^GFP^ CD4+ T cells can be sequentially transduced with dual FOXP3 isoforms. FOXP3KO^GFP^ CD4+ T cells were sequentially transduced with FOXP3FL (NGFR) and FOXP3Δ2 (EGFR). **(A)** Sequential LV transduction strategy of FOXP3 knockout CD4+ T cells. **(B)** Transduction efficiency of FOXP3KO^GFP^ CD4+ T cells (n = 4). **(C)** VCN analysis of FOXP3KO^GFP^ CD4+ T cells transduced with dual FOXP3 isoforms. **(D)** RT-PCR and immunoblotting of FOXP3KO^GFP^ CD4+ T cells transduced with dual FOXP3 isoforms. NS, not significant.

As expression of each isoform alone resulted in suboptimal suppressive function, we next evaluated whether co-expression of both FOXP3 isoforms could improve this activity. For this experiment, we generated additional LV-FOXP3 isoform constructs that replaced the NGFR sequence with the open reading frame for EGFR, thus allowing us to sequentially transduce the FOXP3KO^GFP^ CD4+ T cells and select for cells transduced with both constructs (**Figure 6A**). Specifically, we generated FOXP3KO^GFP^ CD4+ T cells expressing 1) FOXP3FL only (FOXP3FL-NGFR+ FOXP3FL-EGFR); 2) FOXP3Δ2 only (FOXP3Δ2-NGFR+ FOXP3Δ2-EGFR); 3) FOXP3FL-NGFR + FOXP3Δ2-EGFR, and control; and 4) NGFR + EGFR (**Figure 6B**). In all cases, the VCN was almost identical (FOXP3FL/FL 4.08 ± 0.07 copy/cell, FOXP3Δ2/Δ2 4.09 ± 0.13 copy/cell, FOXP3FL/Δ2 4.22 ± 0.10 copy/cell and NGFR/EGFR 3.92 ± 0.12 copy/cell, MEAN±SEM, n = 4) (**Figure 6C**). Furthermore, RT-PCR and immunoblotting showed similar expression levels of mRNA and protein for each FOXP3 isoform (**Figure 6D**).

**Figure 7:**
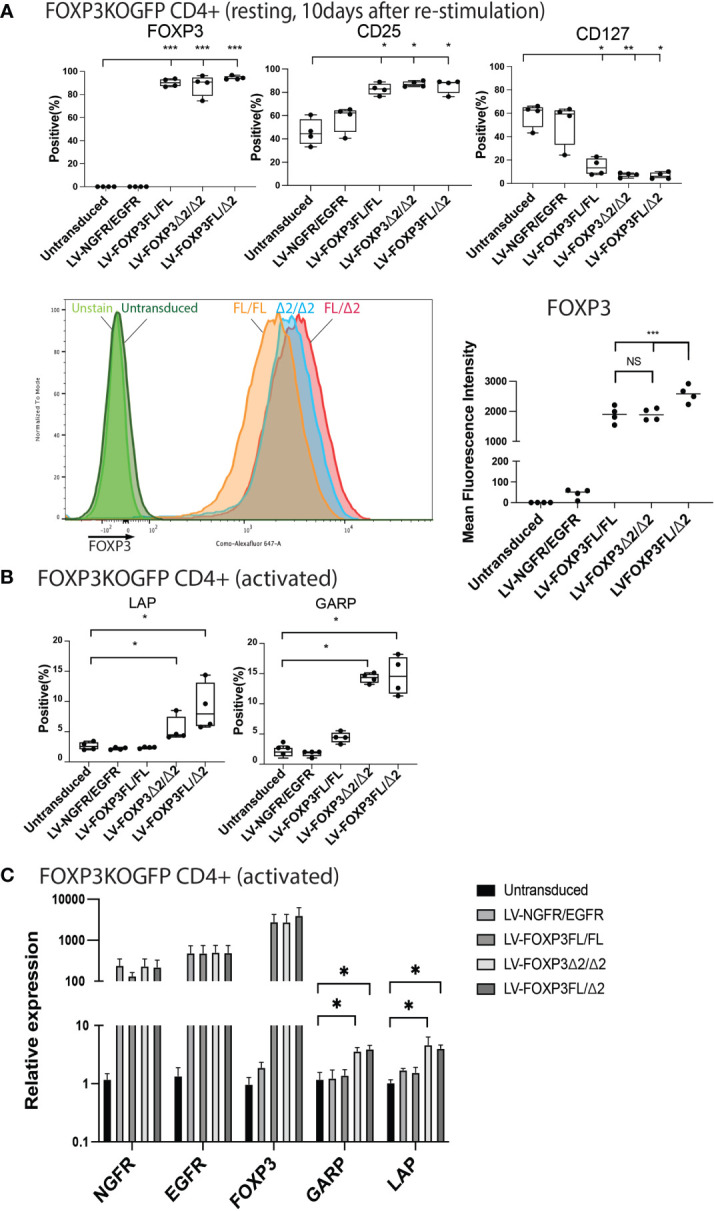
Dual transduction of different isoforms provides Treg-like phenotype in FOXP3KO^GFP^ CD4+ T cells. FOXP3KO^GFP^ CD4+ T cells were sequentially transduced with FOXP3FL(NGFR)/FOXP3FL(EGFR), FOXP3δ2(NGFR)/FOXP3δ2(EGFR), and FOXP3FL(NGFR)/FOXP3δ2(EGFR). **(A, B)** FACS staining of FOXP3KO^GFP^ CD4+ T cells transduced with dual FOXP3 isoforms (n = 4). **(C)** qPCR analysis of FOXP3KO^GFP^ CD4+ T cells transduced with dual FOXP3 isoforms (n = 4). NS, not significant, *P < 0.05, **P < 0.01, ***P < 0.001.

We next analyzed various Treg-cell features and functions under all four transduction conditions, and the untransduced control and data are summarized in **Table 1**. Transduction of FOXP3FL only and FOXP3Δ2 only resulted in cells positive for FOXP3 expression. Further, and together with FOXP3FL + FOXP3Δ2, FOXP3FL only and FOXP3Δ2 only conferred Treg-like phenotypes (CD25high CD127low FOXP3 positive) to the transduced FOXP3KO^GFP^ CD4+ T cells. Double transduction of FOXP3FL + FOXP3Δ2 led to a significantly higher overall FOXP3 protein expression, when compared with double transduction and expression of each isoform alone (**Figure 7A**). The control NGFR+EGFR transduced cells were indistinguishable from the untransduced cells, confirming the need for FOXP3 expression in the development of Treg features. Interestingly, LAP and GARP expression was significantly higher in the FOXP3Δ2 only and FOXP3FL + FOXP3Δ2-transduced cells (**Figure 7B**) when compared with FOXP3FL only or control conditions, confirming the requirement for FOXP3Δ2 for the expression of these proteins (**Figures S6A, B**). LAP and GARP mRNA expression, confirmed by qPCR, was significantly higher in the FOXP3Δ2 only and FOXP3FL + FOXP3Δ2-transduced cells. However, in contrast to the protein expression, FOXP3 mRNA expression was similar in all the three conditions (FOXP3FL only, FOXP3Δ2 only, and FOXP3FL + FOXP3Δ2) (**Figure 7C**).

**Figure 8:**
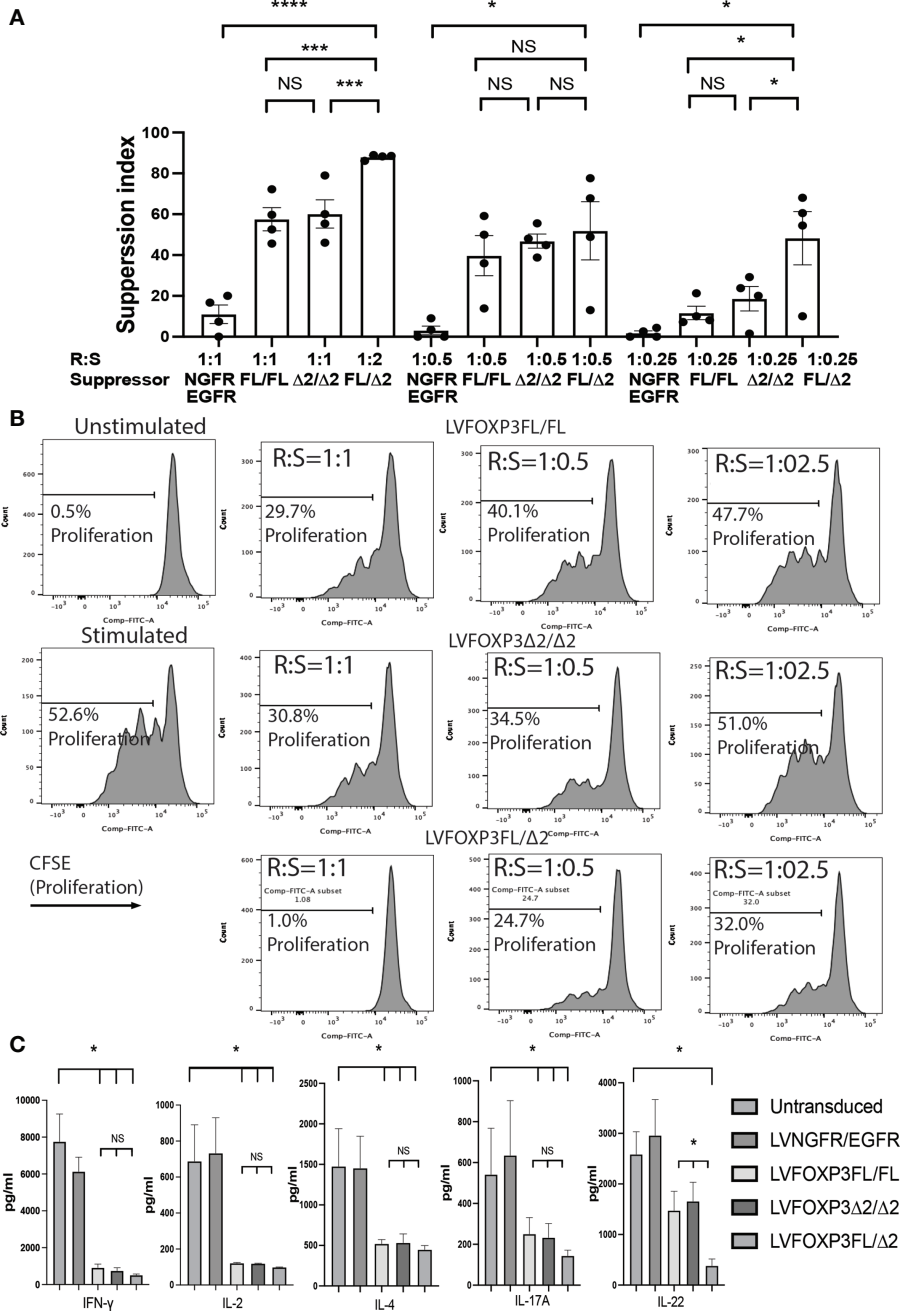
Dual transduction of different isoforms provides Treg-like function in FOXP3KO^GFP^ CD4+ T cells. **(A, B)** Suppressive function of FOXP3KO^GFP^ CD4+ T cells transduced with dual FOXP3 isoforms (n = 4). **(C)** Cytokine production profile of FOXP3KO^GFP^ CD4+ T cells transduced with dual FOXP3 isoforms (n = 4). NS, not significant, *P < 0.05, ***P < 0.001, ****P < 0.0001.

Finally, we analyzed the suppressive function induced by the FOXP3KO^GFP^ CD4+ T cells sequentially transduced with either FOXP3FL, FOXP3Δ2, or FOXP3FL+FOXP3δ2. Strikingly, we found that FOXP3KO^GFP^ CD4+ T cells transduced with FOXP3FL+FOXP3Δ2 had a significantly higher suppressive function (suppression index FOXP3FL/Δ2 88.0 ± 0.6 (R:S = 1:1), MEAN±SEM, n = 4) when compared to double transduction of FOXP3FL only or FOXP3Δ2 only (suppression index FOXP3FL/FL 57.5 ± 5.7 (R:S = 1:1), FOXP3Δ2/Δ2 60.1 ± 6.9 (R:S = 1:1), MEAN ± SEM, n = 4), or controls (**Figures 8A, B** **)**. Importantly, this level of suppression was comparable to that observed with expanded WT Tregs (suppression index expanded WT Tregs 89.9 ± 1.0 (R:S = 1:1), MEAN ± SEM, n = 4) (**Figure S4B**), supporting our hypothesis that expression of both isoforms is required for optimal Treg-suppressive activities. All three transduction conditions, FOXP3FL only, FOXP3Δ2 only, and FOXP3FL+FOXP3Δ2, led to comparable and significant reductions in IL-2, IL-4, and IFN-γ production (**Figure 8C**), when compared to NGFR+EGFR-transduced or untransduced activated FOXP3KO^GFP^ CD4+ T cell controls, suggesting that the expression of either isoform is sufficient, a view that is supported by our single transduction findings (**Table 1**). Moreover, when compared to transduction of FOXP3FL or FOXP3Δ2 only, FOXP3FL+FOXP3Δ2 led to a significant reduction in IL-22 production and a visible, although not statistically significant, reduction of IL-17A, suggesting that complementary activities of both isoforms are required to reduce the expression of IL-17A and IL-22. Overall, these data support the view that optimal suppressive function requires the expression of both FOXP3FL and FOXP3Δ2, suggesting that each FOXP3 isoform has distinct and key roles in conferring Treg-mediated suppression. In addition, the data suggest that while either isoform can induce a reduction in IL-2, IL-4, and IFN-γ production, both isoforms are required to reduce IL-17A and IL-22. We conclude that simultaneous expression of both FOXP3FL and FOXP3Δ2 is required for optimal overall FOXP3 protein levels, acquisition of Treg-like phenotypes, and Treg-suppressive functions.

## Discussion

Human Tregs predominantly express FOXP3 as two isoforms, FOXP3FL and FOXP3Δ2 (16). While some functional similarities and differences of each FOXP3 isoform have been determined, a conclusive analysis of their respective roles in the maintenance of overall FOXP3 protein levels and the impact of each on the induction of Treg suppressive functions has not been clarified (13).

The creation of primary CD4+ T cells in which the endogenous *FOXP3* gene is disrupted, followed by LV-mediated transfer of FOXPFL and/or FOXP3Δ2, has made it possible to dissect roles for each isoform, in the absence of genomic *FOXP3* expression.

Using this system, we show that LV-mediated transfer of both FOXPFL and FOXP3Δ2, and not each alone, leads to the optimal acquisition of a Treg-like phenotype and suppressive functions in transduced FOXP3KO^GFP^ CD4+ T cells *in vitro*. The suppressive activity achieved when both isoforms are present is comparable to that of expanded WT Tregs. Further, the expression of either isoform alone or the simultaneous expression of both leads to comparable and significant reductions in the production of hallmark cytokines IL-2, IL-4, and IFN-γ production, indicating that either FOXP3 isoform is sufficient for this reduction. In contrast, only the co-expression of FOXP3FL+ FOXP3Δ2 leads to a significant reduction in IL-22 and IL-17A production, suggesting that complementary activities of both isoforms are required to reduce the expression of these pro-inflammatory cytokines. These data support several studies that have analyzed Tregs from patients with non-monogenic autoimmune and inflammatory diseases including psoriasis (33), rheumatoid arthritis (34–37), inflammatory bowel disease (38), and coronary artery disease (39) that have demonstrated an aberrant increase in FOXP3FL relative to FOXP3Δ2, suggesting that FOXP3Δ2, or a fine balance between the two isoforms, might be important to maintain the physiological regulatory function and stability of Tregs. Previous work has also shown that FOXP3Δ2 lacks the binding sites to interact with transcriptional regulators, Signal Transducer and Activator of Transcription (STAT)-3, and retinoic acid receptor-related orphan nuclear receptor (ROR) family members, RORγT and RORα. Zhou and colleagues found that loss of RORγT binding leads to an increased expression of IL-17A, a precursor to T helper (Th) 17 lineage development, suggesting a role for regulated FOXP3Δ2 expression in determining Treg/Th17 fate lineages (40). The data we present here support these findings.

Additional FOXP3 isoform-specific expression data were published by Joly et al., who showed that FOXP3Δ2, but not FOXPFL, induces transcription of GARP, a key mediator of the suppressive activities of Tregs (28–31, 41). Our data also suggest that the expression of GARP, as well as LAP, is preferentially induced by FOXP3Δ2 isoform transduction (alone or co-transduced with FOXP3FL). Wang and colleagues showed that GARP-expressing Tregs have a more potent suppressive function than those that do not express GARP (29). Furthermore, Edwards et al. found that GARP-expressing Tregs have reduced IL-17 production capacity compared to non-GARP-expressing Tregs. At the same time, they showed that expression of GARP inhibits Th17 differentiation and promotes Treg differentiation in the presence of IL-2 (30). Finally, Gandhi et al. have shown that, together with GARP, LAP is associated with a suppressive function of Tregs (42). Importantly, however, despite a role for FOXP3Δ2 in induction of GARP, and a clear function for GARP in Treg-suppressive activities, we observe the best suppressive function when the FOXP3KO^GFP^ CD4+ T cells co-express both isoforms, supporting a role for both FOXP3FL and FOXP3Δ2 in mediating optimal Treg-suppressive function. Altogether, these previous observations and our present data imply that the presence of both FOXP3 isoforms can convert FOXP3KO^GFP^ CD4+ T cells into more suppressive Treg-like cells with a higher expression of GARP and reduced production of IL-17A.

To date, *FOXP3* engineering strategies including gene transfer and gene editing to restore protein expression have exclusively focused on FOXP3FL (43), a strategy that has been effective following different approaches (18, 25, 26, 44, 45). Our approach of autologous gene editing in hematopoietic stem cells (HSC), by which FOXP3FL cDNA is inserted at the endogenous *FOXP3* initiation codon using CRISPR/Cas9, uniquely allows the expression of the inserted FOXP3FL cDNA to be expressed using the endogenous *FOXP3* initiation codon and regulated by physiologic, developmental, and lineage-specific signals. Results obtained so far, in normal or IPEX patient mature Treg and Teff cells *in vitro*, demonstrated that this method results in the restoration of normal FOXP3 expression in Teff cells, but only 50% of normal expression in Tregs and suboptimal Treg-suppressive activity. Similarly, the T cell compartment obtained upon *in vivo* differentiation of FOXP3FL-edited HSCs contains a lower than expected frequency of FOXP3-expressing Tregs, when compared to unedited HSCs. These data suggest that the expression of FOXP3FL alone might not be sufficient to restore all the endogenous FOXP3 functions required for normal T cell ontogeny and a normal immune system development (18). However, we also cannot discount the possibility that, in addition to the absence of FOXP3Δ2, the endogenous FOXP3 introns that are lacking in the FOXP3 cDNA might contain key regulatory elements required for optimal FOXP3 expression (46).

As an alternative strategy, it was shown that dual transduction of FOXP3FL and Helios isoforms could convert CD4+ T cells into Treg-like cells (47), indicating the presence of additional factors that can facilitate the Treg conversion by FOXP3FL gene transfer alone.

Dual transduction of both FOXP3 isoforms could fully restore the suppressive function of FOXP3 knockout CD4+ T cells. Compared to the single transduction, FOXP3 knockout CD4+ T cells transduced with both isoforms acquired a Treg-like phenotype (CD25^high^ CD127^low^ FOXP3^high^). *In vitro* suppressive function was statistically higher in FOXP3 knockout CD4+ T cells transduced with dual isoforms compared to those transduced with either FOXP3FL or FOXP3Δ2 alone. These data indicate that both FOXP3FL and FOXP3Δ2 are required to convert FOXP3 knockout CD4+ T cells into functional Tregs.

Here we have successfully shown that FOXP3FL and FOXP3Δ2 have synergistic, non-overlapping effects in the conversion of FOXP3KO^GFP^ CD4+ T cells into Treg-like cells, including the expression of molecules that have a direct role in the suppression and inhibition of inflammatory cytokines such as IL-17 and IL-22, which could not be inhibited by either isoform alone. In addition, the FOXP3FL/Δ2-transduced cells showed the highest level of suppression of proliferation of activated Teff cells. Our data also exclude the possible contribution of endogenous FOXP3 expression in the generation of the Treg function we observe and at the same time exclude that these findings are simply due to the over-transduction since the VCN in the different cell type is comparable. Our present observations highlight the synergistic effects between FOXP3FL and FOXP3Δ2 and suggest that the restoration of both FOXP3FL or FOXP3Δ2 may be required to provide fully functional Tregs. Our findings imply that restoration of both FOXP3 isoforms leads to a more physiological rescue of Treg-cell features and functions, therefore supporting further investigation as a molecular therapeutic approach for the correction of *FOXP3*-deficient T cells and Tregs.

In conclusion, we have shown that complementary functional regulatory activity is conferred by FOXP3FL and FOXP3Δ2 isoform gene transfer in FOXP3KO^GFP^ CD4+ T cells. As such, maintenance of the expression of both isoforms must be further investigated in future gene editing strategies aimed at fully restoring FOXP3 functions in *FOXP3*-deficient cells such as those in IPEX syndrome patients.

## Data Availability Statement

The raw data supporting the conclusions of this article will be made available by the authors, without undue reservation.

## Ethics Statement

Ethical review and approval were not required for the study on human participants in accordance with the local legislation and institutional requirements. The patients/participants provided their written informed consent to participate in this study.

## Author Contributions

YS conducted the experiments and wrote the manuscript. JL conducted the experiments and wrote the manuscript. EL contributed to experimental development. RP contributed to the experimental design and manuscript editing and writing. MGR and RB designed the experiments and reviewed the manuscript. All authors contributed to the article and approved the submitted version.

## Funding

This work was supported by the Stanford Maternal and Child Health Research Institute Faculty Scholar Grant to RB. YS was supported by the California Institute for Regenerative Medicine (CLIN1 11591 to RB).

## Conflict of Interest

The authors declare that the research was conducted in the absence of any commercial or financial relationships that could be construed as a potential conflict of interest.

## Publisher’s Note

All claims expressed in this article are solely those of the authors and do not necessarily represent those of their affiliated organizations, or those of the publisher, the editors and the reviewers. Any product that may be evaluated in this article, or claim that may be made by its manufacturer, is not guaranteed or endorsed by the publisher.

## Supplementary Material

Supplementary Figure 1 HEK293T/17 cells transduced with FOXP3 isoforms **(A)** Lentiviral vector construct. Cytomegalovirus minimal promoter (mCMV), Human elongation factor-alpha promoter (EF-1α) **(B)** Expression of marker genes (NGFR and EGFR) **(C)** Expression of FOXP3. Representative FACS dot-plot of HEK293T/17 cells transduced with FOXP3 isoforms. **(D)** Expression of marker genes (NGFR and EGFR) **(E)** Expression of FOXP3.

Supplementary Figure 2 Representative FACS dot-plot of MT-2 cells transduced with FOXP3 isoforms. **(A)** Expression of marker genes (NGFR and EGFR) **(B)** Expression of FOXP3 and GFP.

Supplementary Figure 3 Wild-type MT-2 cells transduced with FOXP3 isoforms. **(A)** LV transduction strategy of WT MT-2 cells **(B)** FOXP3 expression of WT MT-2 cells transduced with FOXP3 isoforms **(C)** RT-PCR and immunoblotting of WT MT-2 cells transduced with FOXP3 isoforms.

Supplementary Figure 4 Phenotype and suppressive function of expanded Tregs. **(A)** FACS staining of expanded Tregs from FOXP3WT CD4+ T cells (n=4) **(B)** Suppressive function of expanded Tregs from FOXP3WT CD4+ T cells (n=4).

Supplementary Figure 5 Single transduction of different isoforms provides Treg-like phenotype in FOXP3KO^GFP^ CD4+ T cells. FACS staining of FOXP3KO^GFP^ CD4+ T cells transduced with single FOXP3 isoforms (n=4) shown by representative **(A)** dot-plot and **(B)** histograms.

Supplementary Figure 6 Dual transduction of different isoforms provides Treg-like phenotype in FOXP3KO^GFP^ CD4+ T cells. FACS staining of FOXP3KO^GFP^ CD4+ T cells transduced with dual FOXP3 isoforms (n=4) shown by representative **(A)** dot-plot and **(B)** histograms.
