# Supplementary figures and images for: Co-Expression of FOXP3FL and FOXP3Δ2 Isoforms Is Required for Optimal Treg-Like Cell Phenotypes and Suppressive Function
Source: Front Immunol. 2021 Oct 19;12:752394. doi: 10.3389/fimmu.2021.752394 (PMC8560788; doi:10.3389/fimmu.2021.752394)

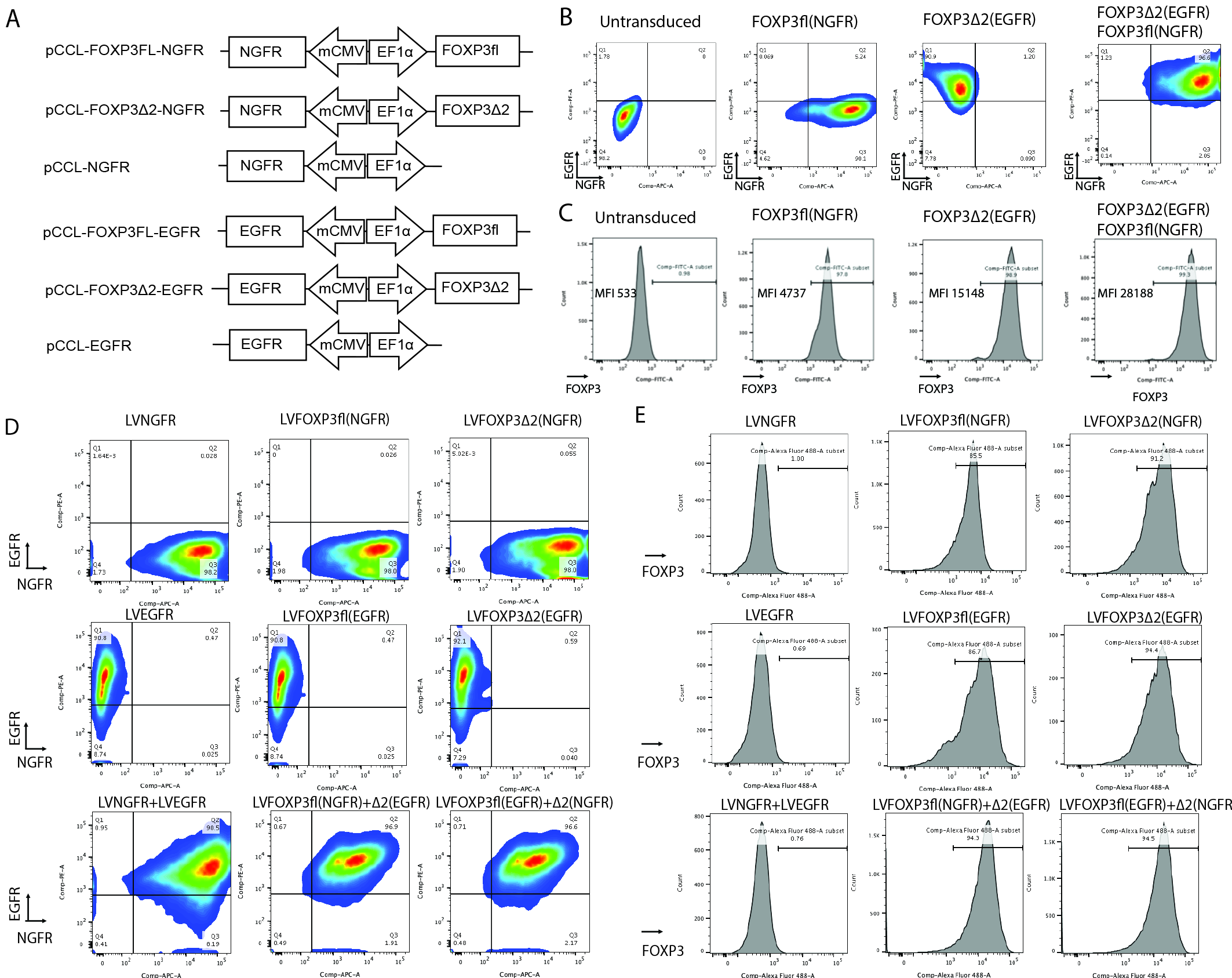

Supplement: Supplementary Figure 1 — HEK293T/17 cells transduced with FOXP3 isoforms (A) Lentiviral vector construct. Cytomegalovirus minimal promoter (mCMV), Human elongation factor-alpha promoter (EF-1α) (B) Expression of marker genes (NGFR and EGFR) (C) Expression of FOXP3. Representative FACS dot-plot of HEK293T/17 cells transduced with FOXP3 isoforms. (D) Expression of marker genes (NGFR and EGFR) (E) Expression of FOXP3. [file Image_1.tif]

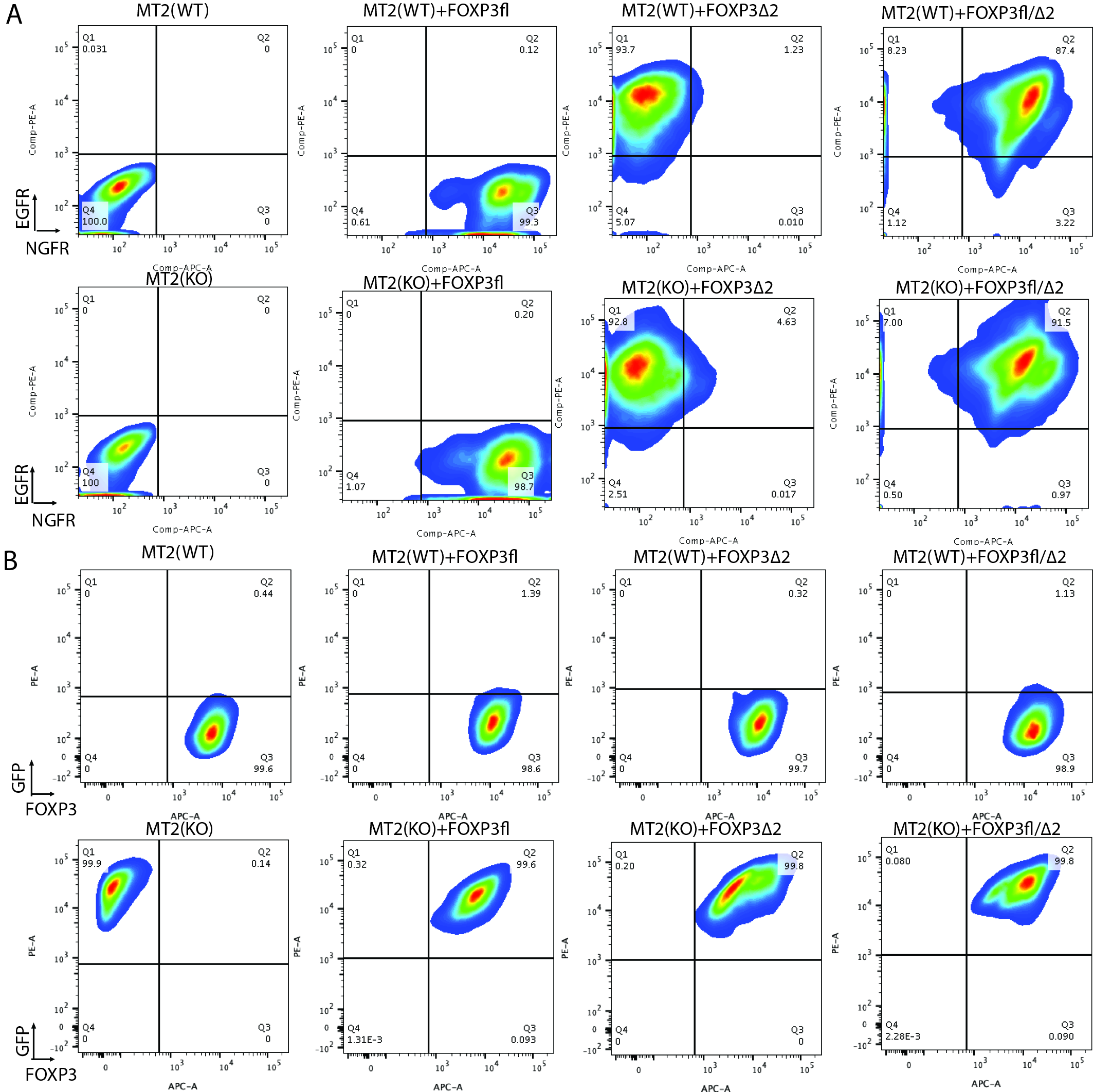

Supplement: Supplementary Figure 2 — Representative FACS dot-plot of MT-2 cells transduced with FOXP3 isoforms. (A) Expression of marker genes (NGFR and EGFR) (B) Expression of FOXP3 and GFP. [file Image_2.tif]

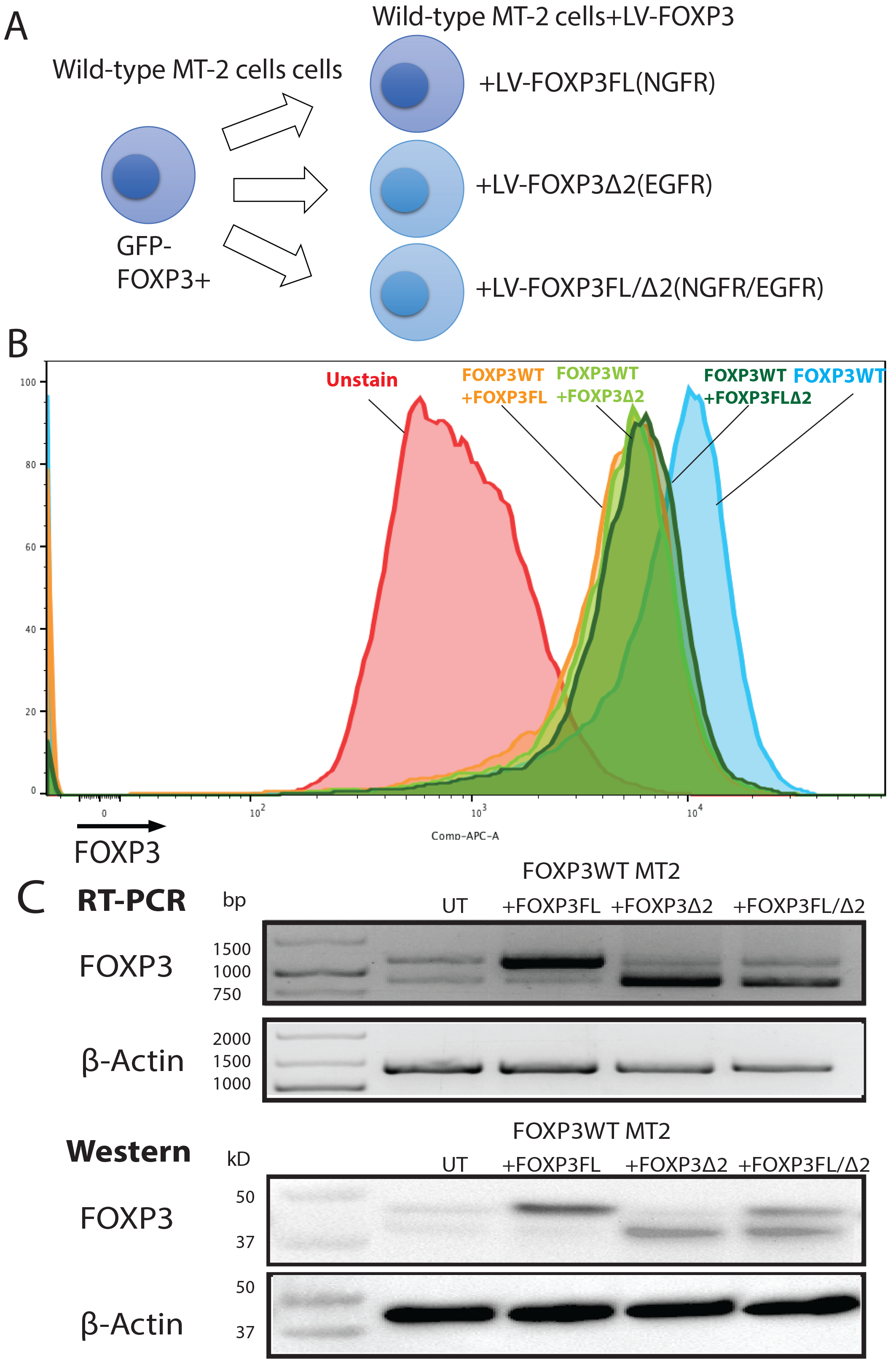

Supplement: Supplementary Figure 3 — Wild-type MT-2 cells transduced with FOXP3 isoforms. (A) LV transduction strategy of WT MT-2 cells (B) FOXP3 expression of WT MT-2 cells transduced with FOXP3 isoforms (C) RT-PCR and immunoblotting of WT MT-2 cells transduced with FOXP3 isoforms. [file Image_3.tif]

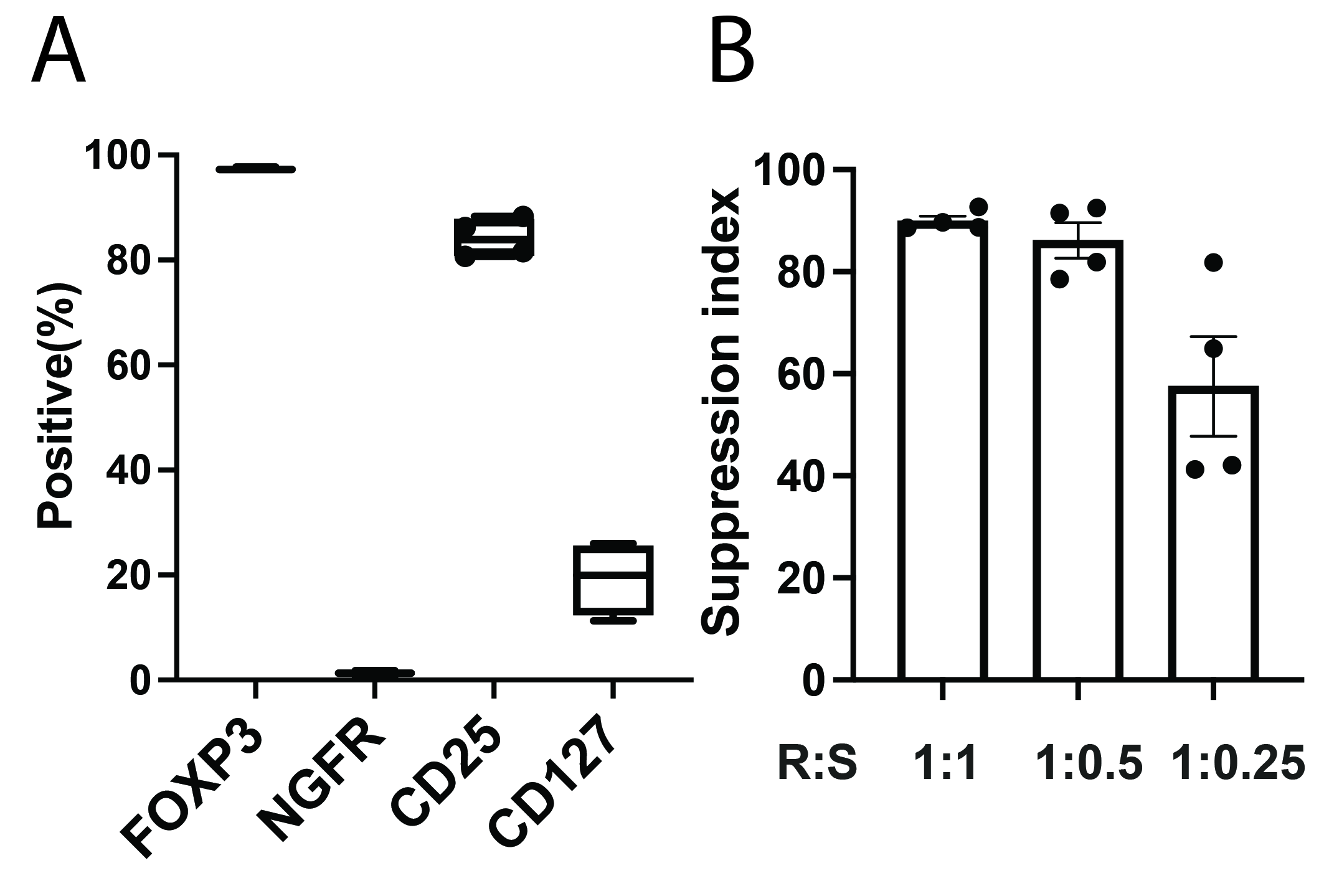

Supplement: Supplementary Figure 4 — Phenotype and suppressive function of expanded Tregs. (A) FACS staining of expanded Tregs from FOXP3WT CD4+ T cells (n=4) (B) Suppressive function of expanded Tregs from FOXP3WT CD4+ T cells (n=4). [file Image_4.tif]

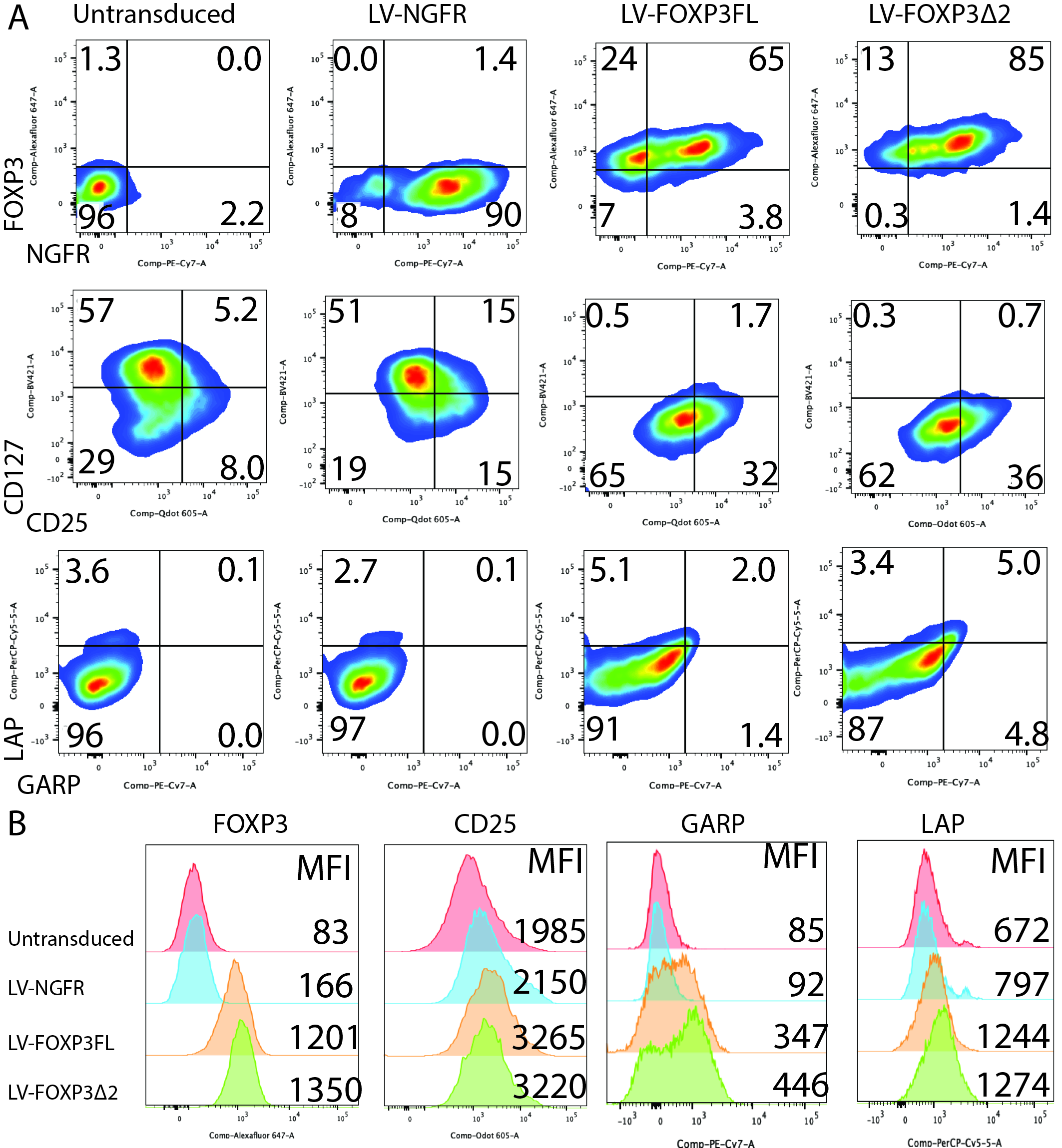

Supplement: Supplementary Figure 5 — Single transduction of different isoforms provides Treg-like phenotype in FOXP3KOGFP CD4+ T cells. FACS staining of FOXP3KOGFP CD4+ T cells transduced with single FOXP3 isoforms (n=4) shown by representative (A) dot-plot and (B) histograms. [file Image_5.tif]

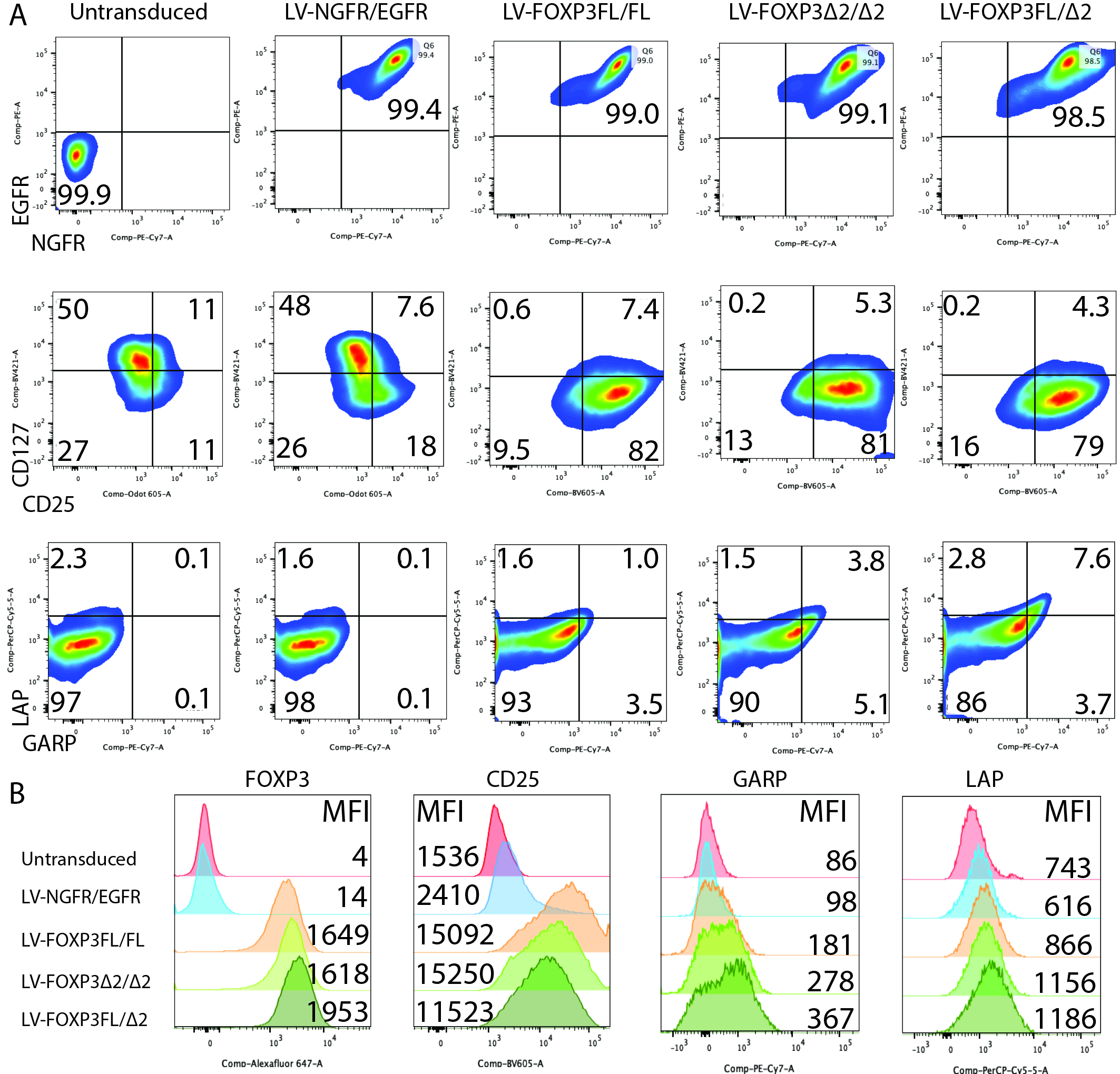

Supplement: Supplementary Figure 6 — Dual transduction of different isoforms provides Treg-like phenotype in FOXP3KOGFP CD4+ T cells. FACS staining of FOXP3KOGFP CD4+ T cells transduced with dual FOXP3 isoforms (n=4) shown by representative (A) dot-plot and (B) histograms. [file Image_6.tif]
